# Propolis Reduces Inflammation and Dyslipidemia Caused by High-Cholesterol Diet in Mice by Lowering ADAM10/17 Activities

**DOI:** 10.3390/nu16121861

**Published:** 2024-06-13

**Authors:** Ertugrul Yigit, Orhan Deger, Katip Korkmaz, Merve Huner Yigit, Huseyin Avni Uydu, Tolga Mercantepe, Selim Demir

**Affiliations:** 1 Department of Medical Biochemistry, Faculty of Medicine, Karadeniz Technical University, 61080 Trabzon, Turkey; odeger@ktu.edu.tr; 2 Department of Nutrition and Dietetics, Faculty of Health Science, Karadeniz Technical University, 61080 Trabzon, Turkey; katip.korkmaz@ktu.edu.tr (K.K.); selim-demir@hotmail.com (S.D.); 3 Department of Medical Biochemistry, Faculty of Medicine, Recep Tayyip Erdogan University, 53000 Rize, Turkey; merve.huner@erdogan.edu.tr; 4 Department of Medical Biochemistry, Faculty of Medicine, Samsun University, 55080 Samsun, Turkey; huseyin.uydu@samsun.edu.tr; 5 Department of Histology and Embryology, Faculty of Medicine, Recep Tayyip Erdogan University, 53000 Rize, Turkey; tolga.mercantepe@erdogan.edu.tr

**Keywords:** ADAM10, ADAM17, ApoE^-/-^, atherosclerosis, propolis

## Abstract

Atherosclerosis is one of the most important causes of cardiovascular diseases. A disintegrin and metalloprotease (ADAM)10 and ADAM17 have been identified as important regulators of inflammation in recent years. Our study investigated the effect of inhibiting these enzymes with selective inhibitor and propolis on atherosclerosis. In our study, C57BL/6J mice (*n* = 16) were used in the control and sham groups. In contrast, ApoE^-/-^ mice (*n* = 48) were used in the case, water extract of propolis (WEP), ethanolic extract of propolis (EEP), GW280264X (GW-synthetic inhibitor), and solvent (DMSO and ethanol) groups. The control group was fed a control diet, and all other groups were fed a high-cholesterol diet for 16 weeks. WEP (400 mg/kg/day), EEP (200 mg/kg/day), and GW (100 µg/kg/day) were administered intraperitoneally for the last four weeks. Animals were sacrificed, and blood, liver, aortic arch, and aortic root tissues were collected. In serum, total cholesterol (TC), triglycerides (TGs), and glucose (Glu) were measured by enzymatic colorimetric method, while interleukin-1β (IL-1β), paraoxonase-1 (PON-1), and lipoprotein-associated phospholipase-A2 (Lp-PLA2) were measured by ELISA. Tumor necrosis factor-α (TNF-α), interferon-γ (IFN-γ), myeloperoxidase (MPO), interleukin-6 (IL-6), interleukin-10 (IL-10), and interleukin-12 (IL-12) levels were measured in aortic arch by ELISA and ADAM10/17 activities were measured fluorometrically. In addition, aortic root and liver tissues were examined histopathologically and immunohistochemically (ADAM10 and sortilin primary antibody). In the WEP, EEP, and GW groups compared to the case group, TC, TG, TNF-α, IL-1β, IL-6, IL-12, PLA2, MPO, ADAM10/17 activities, plaque burden, lipid accumulation, ADAM10, and sortilin levels decreased, while IL-10 and PON-1 levels increased (*p* < 0.003). Our study results show that propolis can effectively reduce atherosclerosis-related inflammation and dyslipidemia through ADAM10/17 inhibition.

## 1. Introduction

Cardiovascular diseases are a prevalent health issue worldwide, and unfortunately, their occurrence is on the rise. These diseases are linked to various cardiometabolic risk factors, such as atherosclerosis, dyslipidemia, high blood pressure, high blood sugar levels, increased inflammatory markers, and oxidative stress [1]. Atherosclerosis, a critical factor in the development of cardiovascular disease, is caused by lipid peroxidation, endothelial dysfunction, vascular smooth muscle cells, platelets, and inflammatory mediators [2]. When there is a surplus of oxidized lipid molecules in the subendothelial region, macrophages cannot remove them, leading to lipid accumulation and lipid droplet formation. These foam-like cells, known as foam cells, are essential in developing atherosclerotic plaque and increasing the plaque load [3].

The members of the ADAM (a disintegrin and metalloprotease) family are essential regulators of cytokines during inflammation. They become more prevalent during the development of foam cells and plaque. Of the 21 ADAMs in the mammalian genome, ADAM10 and ADAM17 are the most researched and understood because of their attractive substrates. These substrates include endothelial growth factor ligands, signaling pathway ligands like the NOTCH signaling pathway, and various inflammatory agents such as tumor necrosis factor-α (TNF-α) and interleukins, adhesion molecules, and sortilin. Sortilin is believed to play a crucial role in dyslipidemia. ADAM10/17 is a target molecule candidate in all diseases where inflammation plays a role, especially cancer. Various synthetic inhibitors, such as GW280264X (GW), BMS-561392, and TMI-005, have been developed. The inhibition of these enzymes has been studied in various pathologies [4,5,6]. However, the increasing side effects of synthetic drugs in recent years have led people to return to natural eating habits [7]. As a result, the interest in nutritional therapy and supplements is growing. One of the most important products in this market is bee products.

Propolis is a natural substance rich in polyphenols that honeybees collect from various plants. Its color and aroma vary depending on the plant species, and its chemical composition changes accordingly [8]. Propolis comprises about 50% resins, balsams, waxes, oils, pollen, and other compounds [9,10]. With over 300 chemical components, it has been found to have anti-inflammatory, antioxidant, antiatherogenic, and immunostimulating effects, making it a popular treatment in traditional medicine and apitherapy [11,12]. In addition, preclinical and clinical investigations of various propolis extracts against prevalent diseases, especially obesity and diabetes, are ongoing [13,14,15].

Although propolis has been found to have higher biological activity when dissolved in solvents such as ethanol and methanol, recent studies have focused on extracting it using water, a non-toxic solvent [16,17]. One of the components of propolis, pinocembrin flavonoid, has been shown to inhibit the gene expression and activity of matrix metalloproteinase-9 (MMP-9), which is increased in macrophages induced by lipopolysaccharide [18]. It is believed that other compounds in propolis may also have inhibitory activity against ADAM10/17, which are proteases similar to MMP and ADAMTS. Since the common substrate and inhibitory activity of ADAM proteases with MMP and a disintegrin and metalloprotease thrombospondin (ADAMTS) is known [19] it was thought that many compounds in the content of propolis would show inhibitory activity against ADAM10/17.

This study aimed to establish an experimental model of atherosclerosis in ApoE^-/-^ mice using a high-cholesterol diet (HCD) and to investigate the biochemical and histological effects of inhibiting ADAM10/17 on the development of atherosclerosis. Additionally, the study aimed to evaluate the impact of water and ethanolic propolis extracts on atherosclerosis development and investigate their effectiveness on ADAM10/17 activities in silico and in vivo.

## 2. Materials and Methods

### 2.1. Molecular Docking Studies

In a recent study on propolis content, water and ethanolic extracts were analyzed using HPLC-DAD. The water extract mainly contained chlorogenic acid, caffeic acid, and naringenin polyphenols, while the ethanolic extract contained numerous flavonoids, particularly chrysin, pinocembrin, and galangin [20]. Accordingly, eleven flavonoids found in the propolis structure were included in the in silico study. The study also performed a molecular docking analysis to investigate the interactions of these flavonoids and a synthetic chemical inhibitor with two target proteins—the ADAM10 extracellular domain and the membrane-proximal domain of ADAM17. The crystal structures for these proteins were downloaded from the RCSB Protein Data Bank and were prepared using the BIOVIA DS Visualizer software 4.5. The ligands were retrieved from the PubChem database and converted to a pdb file using the same software. The docking simulations were performed using AutoDock 4.2 software with the Lamarckian genetic algorithm, and the results were analyzed using BIOVIA Discovery Studio Visualizer 2018 [21]. The study ran 100 genetic algorithm runs and analyzed the binding energies of forty-five docked conformations of each ligand against the target proteins.

### 2.2. Chemicals and Diets

DMSO (34869), ethanol (1.11727), Triton x100 (X-100), and PBS (524650-1EA) were obtained from Sigma–Aldrich (St. Louis, MO, USA). GW280264X (AOB3632) with 98% purity was purchased from AOBIOUS (Boston, MA, USA) and dissolved in 10% DMSO. HCD (Clinton/Cybulsky High Cholesterol Rodent Diet With Regular Casein and 1.25% Added Cholesterol, D12108C, Research Diet, New Brunswick, NJ, USA) and control diet (CD) (a low-fat cholesterol-containing diet without cholic acid, D12104C, Research Diet, New Brunswick, NJ, USA) were purchased from Research Diet. Our study used propolis samples collected from various regions of Turkey, for which HPLC-DAD and GC-MS content analyses were performed. Oil red O (ORO) (Merck Millipore, 102419, Darmstadt, Germany), Mayers Hematoxylin (Merck GmbH, Darmstadt, Germany), sortilin primary antibody (ab268864, Abcam, Cambridge, UK), and ADAM10 primary antibodies (1/100, ab227172, Abcam, Cambridge, UK) and secondary antibody (ab205718, Abcam, Cambridge, UK) were used for histological analysis.

### 2.3. Animals and Experimental Groups

The experimental protocol with reference number 2021-45 was approved by the Local Ethics Committee and Animals Research of Karadeniz Technical University. The experiment adhered to the guidelines for the care and use of laboratory animals established by the National Institutes of Health (NIH Publications No. 8023, revised 1978) and the ARRIVE guidelines for reporting experiments involving animals [22,23]. For the DIO mouse experiments, wild-type (*n* = 16, C57BL/6J) mice (RRID: IMSR_JAX:000664) and Apolipoprotein-E knockout (*n* = 48, ApoE^-/-^) mice (RRID: IMSR_JAX:002052) were purchased from Karadeniz Technical University Surgical Research Center (Trabzon, Turkey) at six to eight weeks of age and were housed at a controlled temperature of 22–23 °C with a 12 h light/dark cycle. ApoE^-/-^ mice have high circulating cholesterol levels due to LDL receptor ligand deficiency and are an important model for atherosclerosis studies [24]. The effect size was calculated with the G*Power 3.1. (Kiel, Germany) program. With the entered effect size (0.40), alpha error (0.05), power (0.80), and group number (8) values, the total sample size was found to be 64. WT mice were randomly divided into two experimental groups, while ApoE^-/-^ mice were randomly divided into six experimental groups. Random numbers were generated using Microsoft Excel 2023. While wild-type (WT) mice were used in control and sham groups, ApoE^-/-^ mice were used in case water extract of propolis (WEP), ethanolic extract of propolis (EEP), GW280264X (GW), DMSO, and ethanol groups. The control group was fed with a control diet, and the other groups were fed with HCD for 16 weeks. During the final 28 days of the 16-week period, a single daily dose of GW 100 µg/kg, EEP 200 mg/kg, WEP 400 mg/kg, DMSO 10%, and ethanol 30% was administered through i.p. injection. In addition, the control, sham, and case groups were given 0.9% NaCl via i.p. injection. GW280264 was prepared by dissolution in distilled water containing 10% DMSO. The powdered propolis was incubated in 30% ethanol for EEP and distilled water for WEP at 60 °C and 150 rpm for 24 h with shaking. It was freshly prepared daily by passing through 0.45 and 0.22 µm filters, respectively. During the 16-week diet program, the mice were weighed and body weight (BW) recorded for four weeks. At the end of the dietary protocol, animals were sacrificed by decapitation following anesthesia (80 mg/kg ketamine/10 mg/kg xylazine). Blood samples were collected into gel separation tubes, and sera were obtained and stored at −80 °C until the analysis day. After the aortic arch, aortic root, and liver tissues of the mice were removed, a piece was dissected and reserved for histopathological examinations, and some were stored at −80 °C for biochemical research.

### 2.4. Serum Biochemistry Analysis

Glucose (Glu), triglyceride (TG), total cholesterol (TC), alanine aminotransferase (ALT), aspartate aminotransferase (AST), and blood urea nitrogen (BUN) levels in serum samples obtained from mice were measured in the Beckman Coulter Analyzer AU 5800 (Brea, CA, USA) autoanalyzer available in the Medical Biochemistry Laboratory of Karadeniz Technical University Farabi Hospital.

Serum interleukin (IL)-1β (BostonChem, Boston, MA, USA), phospholipase-A2 (PLA2) (BT-LAB, Shanghai, China), and paraoxonase-1 (PON-1) (BT-LAB, Shanghai, China) levels were measured according to the manufacturer’s protocol using a commercial enzyme-linked immunosorbent assay (ELISA) kit. In each well of the appropriate antibody-coated ELISA kits, 40 µL serum for PLA2 and PON-1, 100 µL serum for IL-1β, and an equal amount of standard were added, and a sandwich ELISA protocol was applied. Colorimetric measurement was performed at 450 nm (Spectra-Max Paradigm, Molecular Devices, Berkshire, UK).

### 2.5. Tissue Preparation

Aortic arch tissues were placed in a solution of 1 mL PBS with 0.01% Triton X-100 buffer. The mixture was homogenized using a homogenizer at 5000 rpm for 90 s on ice. After homogenization, 15 s of sonication (130 Watt, 20 Khz) was applied using a sonicator (VCX500, Sonics-Vibracell, Newtown, PA, USA). The homogenates were centrifuged at 10,000× *g* for 10 min (Allegra^TM^ 64R, Beckman Coulter, Brea, CA, USA), and the supernatant was carefully removed. Protein determination was performed using a commercial bicinchoninic acid kit (BCA1, Sigma-Aldrich, Darmstadt, Germany).

### 2.6. Tissue ADAM10 and ADAM17 Activity Assay

Aortic arch, ADAM10 (ANASPEC, Fremont, CA, USA), and ADAM17 (ANASPEC, CA, USA) activities were determined by commercial kits based on the fluorometric measurement method. Briefly, kinetic measurements were performed using 5-FAM (fluorophore) and QXL™ 520 (quencher)-labeled FRET peptide substrate with excitation/emission = 490 nm/520 nm (Spectra-Max Paradigm, Molecular Devices, Berkshire, UK).

### 2.7. Tissue Inflammatory Mediators

Aortic arch TNF-α (BostonChem, Boston, USA), interferon-γ (IFN-γ) (BostonChem, Boston, USA), IL-6 (BostonChem, Boston, USA), IL-12 (BostonChem, Boston, USA), MPO (BostonChem, Boston, USA), and IL-10 (BostonChem, Boston, USA) levels were measured according to the manufacturer’s protocol using a commercial ELISA kit. An equal amount of supernatant (100 µL) and standard was added to each well of the appropriate antibody-coated ELISA kits, and a sandwich ELISA protocol was applied. Colorimetric measurement was performed at 450 nm (Spectra-Max Paradigm, Molecular Devices, Berkshire, UK).

### 2.8. Histological and Immunohistochemical Analysis in Aortic Root and Liver

Sections of the aortic root and liver tissue were trimmed to a volume of 1.5 cm^3^. They were kept at −80 °C for 10 min, and thick sections of tissue samples of 6–8 µm were taken with a cryostat (Leica 3050S, Leica Biosystems, Nußloch, Germany) at −18 °C using a freezing medium. The sections were stained with ORO in the next step. After staining with ORO, counterstaining was performed with Mayers Hematoxylin. The sections were analyzed and photographed using a digital camera (Olympus DP71, Olympus Corp, Tokyo, Japan) and a light microscope (Olympus BX51, Olympus Corp, Tokyo, Japan). While performing tissue follow-up, sections of the aortic root and liver tissue were taken with a rotary microtome, and serial sections of 2–3 µm thickness (Leica RM2255) were taken on positively charged slides (Superior Marinfield Histobond+). Deparaffinization was performed before immunostaining and using the IHC/ISH instrument (Bond Max, Leica Biosystems, Mount Waverley, Australia), sortilin primary antibody, and ADAM10 primary antibody and incubation with the appropriate secondary antibody. Two blinded histologists evaluated sections of the aortic root and liver tissues, each blinded for 20 different field study groups.

### 2.9. Statistical Analysis

Statistical analyses were performed using the SPSS 23.0 program. The Kolmogorov–Smirnov test was used to check whether the data were normally distributed. ADAM10/17 activities (*p* < 0.05) and BW (*p* < 0.01) changes were assessed for normal distribution and expressed as mean ± SD. Group comparisons were performed using ANOVA and Tukey’s test. The other data were expressed as the median (Q1–Q3) for non-normal distribution. Comparisons between groups were made using the Mann–Whitney U test with Bonferroni correction. Regarding Bonferroni correction, α = 0.05/16 = 0.0031 was determined to have statistical significance. The statistical significance level was accepted as *p* < 0.003. Nonparametric histological data were evaluated using Kruskal–Wallis and Tamhane T2 tests. *p* < 0.05 was considered statistically significant for histological data.

## 3. Results

### 3.1. Propolis Extracts Inhibit ADAM10/17

**Figure 1:**
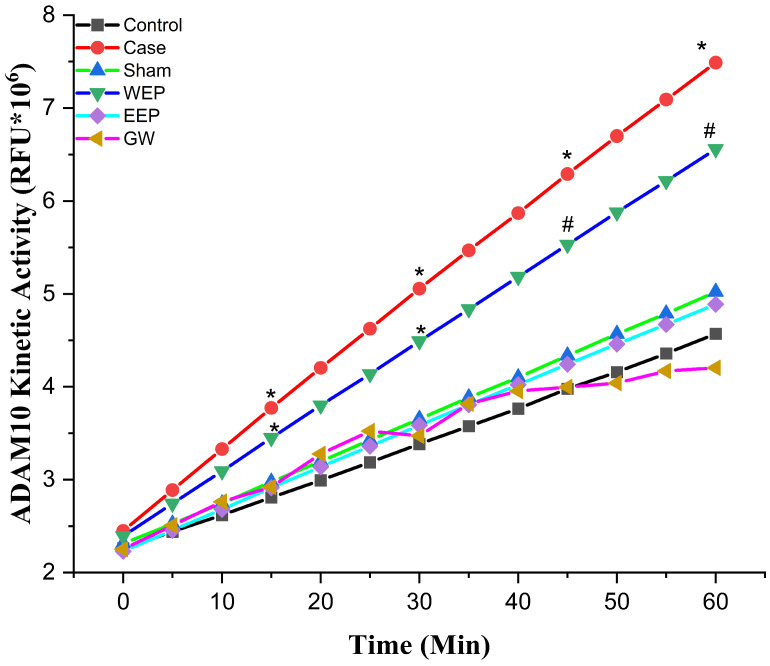
Aortic arch a disintegrin and metalloprotease 10 activity. * statistically significant compared to control (*p* < 0.05); ^#^ statistically significant compared to case (*p* < 0.05) (*n* = 8, One-way ANOVA, the data are expressed as mean).

**Figure 2:**
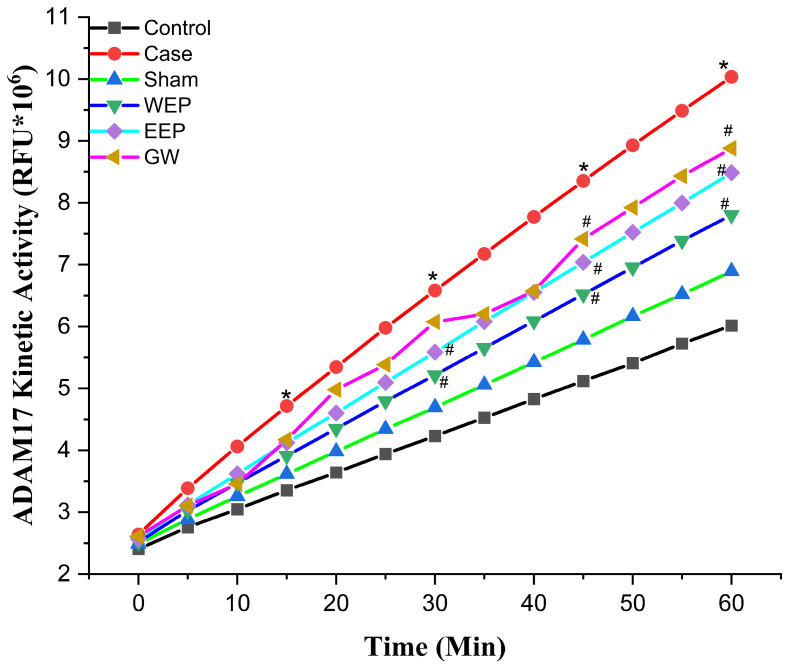
Aortic arch a disintegrin and metalloprotease 17 activity. * statistically significant compared to control (*p* < 0.05); ^#^ statistically significant compared to case (*p* < 0.05) (*n* = 8, One-way ANOVA, the data are expressed as mean).

Molecular docking studies were performed to evaluate the inhibitory potential of examined ligands against two proteins. These simulations gave the predicted protein–ligand binding energy Ki values and identified the potential ligand binding sites. After the successful docking of all the ligands and reference molecules (Table 1) employed in these docking experiments, the results showed significant interactions of the ligands with the target proteins. Fluorometric kinetic measurement results of ADAM10/17, whose activity is increased in atherosclerosis in the aortic arch, are given in Figure 1 and Figure 2, respectively. ADAM10/17 activity was statistically significantly lower in the WEP, EEP, and GW groups than in the case group (*p* < 0.05). 

### 3.2. Propolis Extracts and GW280264X Have Antiobesity Effects

**Figure 3:**
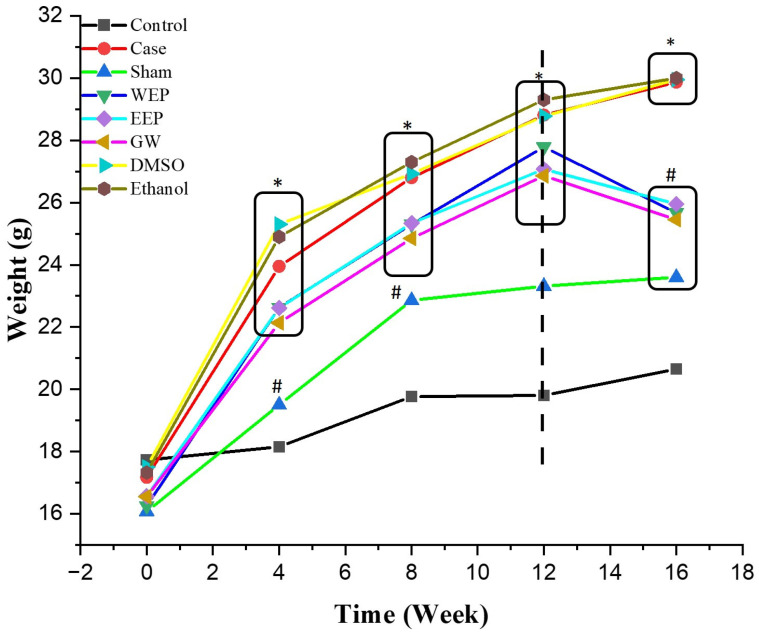
Experimental groups’ body weight (BW) changes. Black dashed line: start of injection. * statistically significant compared to control (*p* < 0.01); ^#^ statistically significant compared to case (*p* < 0.01) (*n* = 8, One-way ANOVA, the data are expressed as mean).

Figure 3 displays the outcomes of the 16-week experimental protocol. At the onset of the study, there was no noteworthy distinction in BW across the different groups (*p* > 0.05). However, the WEP, EEP, GW, DMSO, and ethanol groups administered with HCD had substantially greater BW at the 4th, 8th, and 12th weeks when compared to the sham and control groups (*p* < 0.01). There was no considerable difference among these groups (*p* > 0.01). Towards the end of the 8th, 12th, and 16th weeks, the body weight of the sham group was considerably higher than the control group (*p* < 0.01). After the treatment, during the last four weeks of the experimental protocol, the BW of the animals in the WEP, EEP, and GW groups was notably lower (*p* < 0.01) than the case, DMSO, and ethanol groups but still considerably higher than the control group (*p* < 0.01).

### 3.3. Propolis Extracts and GW280264X Reduce Dyslipidemia and Hyperglycemia

The Glu, TG, TC, ALT, AST, and BUN levels were measured in the sera of different experimental groups. The TC and TG levels were significantly lower in the WEP, EEP, and GW groups as compared to the case group (*p* < 0.003) and higher than the control group (*p* < 0.003). AST was noted as one of the parameters used to evaluate the liver and kidney functions of the extracts. The AST levels were significantly higher in the sham, EEP, and ethanol groups than in the control and case groups (*p* < 0.003). The Glu values were significantly lower in the EEP and GW groups than in the control group (*p* < 0.003). However, there was no significant difference between the groups in BUN and ALT (except WEP) measurements (*p* > 0.003).

### 3.4. Propolis Extracts and GW280264X Have an Antiatherogenic Function

**Figure 4:**
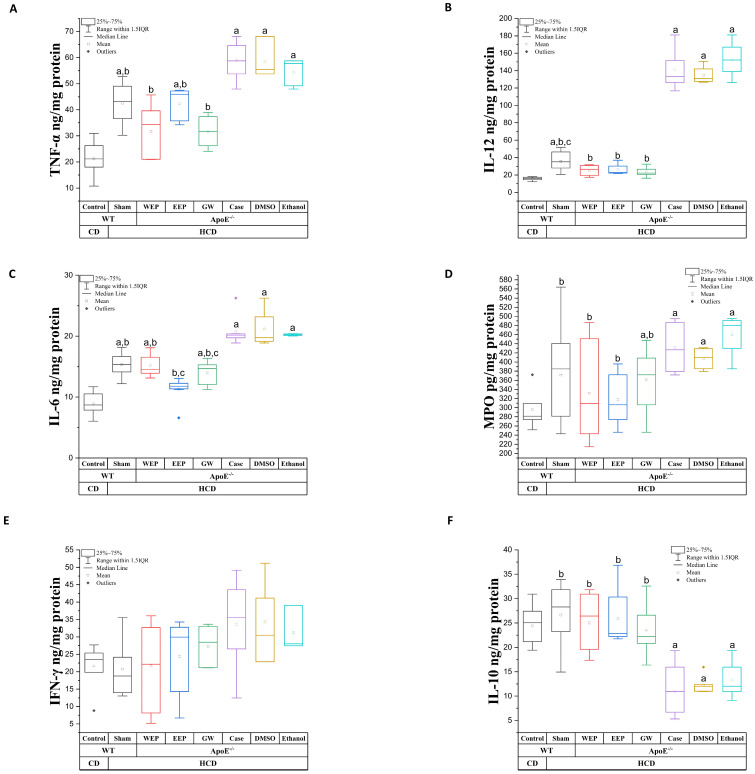
Aortic arch cytokine levels. (**A**) TNF-α levels, (**B**) IL-12 levels, (**C**) IL-6 levels, (**D**) MPO levels, (**E**) IFN-γ levels, (**F**) IL-10 levels. ^a^ statistically significant compared to control (*p* < 0.003); ^b^ statistically significant compared to case (*p* < 0.003); ^c^ statistically significant compared to WEP (*p* < 0.003); (*n* = 8, Mann–Whitney U, the data are expressed as median (Q1–Q3). WT: wild-type mice, ApoE^-/-^: Apolipoprotein-E knockout mice, CD: control diet, HCD: high-cholesterol diet. Gray: Control and Sham (C57BL-6J), Red (ApoE^-/-^, 400 mg/kg WEP), Blue (ApoE^-/-^, 200 mg/kg EEP), Green (ApoE^-/-^, 100 µg/kg GW), Purple (ApoE^-/-^, Case), Brown (ApoE^-/-^, 10% DMSO), Turquoise (ApoE^-/-^, 30% Ethanol).

IL-1β and PLA2 levels, which are atherogenic parameters measured in serum to evaluate inflammation in atherosclerosis, are given in Table 2. IL-1β and PLA2 levels were statistically significantly lower in the WEP, EEP, and GW groups compared to the case group (*p* < 0.003). WEP reduced IL-1β to control levels, just as EEP decreased PLA2 to control levels (*p* > 0.003). TNF-α (Figure 4A), IL-12 (Figure 4B), IL-6 (Figure 4C), MPO (Figure 4D), and IFN-γ (Figure 4E) levels of atherogenic cytokines measured to evaluate inflammation in atherosclerosis in the aortic arch are given in Figure 4.

TNF-α, IL-6, IL-12, and MPO levels were statistically significantly lower in WEP, EEP, and GW groups compared to the case group (*p* < 0.003). Furthermore, WEP, EEP, and GW IL-12 levels decreased to control group levels, just as WEP and GW TNF-α levels decreased to control group levels (*p* > 0.003). Although the WEP, EEP, and GW groups had lower IFN-γ levels than the case group, no statistically significant difference was found (*p* > 0.003). The antiatherogenic cytokine IL-10 levels measured to evaluate inflammation in atherosclerosis in the aortic arch are given in Figure 4F. IL-10 levels were statistically significantly higher in the WEP, EEP, and GW groups than in the case group (*p* < 0.003). In addition, there was no statistically significant difference between the WEP, EEP, and GW groups and the control group (*p* > 0.003). Antioxidant enzyme PON-1 levels measured in serum to evaluate oxidative stress in atherosclerosis are given in Table 2. While the PON-1 levels were statistically significantly higher in the WEP and EEP groups compared to the case group (*p* < 0.003), no statistically significant difference was found between the GW group and the case group (*p* > 0.003).

### 3.5. Propolis Extracts and GW280264X Reduce Atherosclerotic Plaque Burden and Adiposity in the Aortic Root

**Figure 5:**
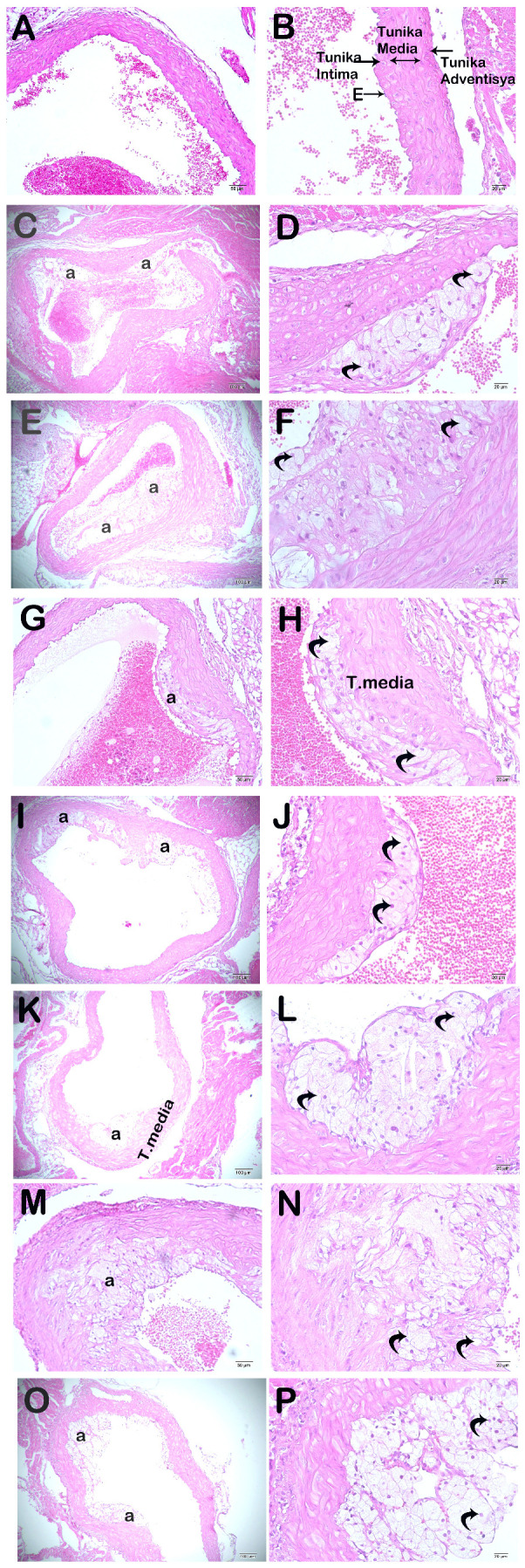
Representative light microscopic image of sections of aortic root tissue stained with H&E. (**A**) (×20) and (**B**) (×40): Control group: In the aortic root tissue sections of the control group, it is observed that the endothelium and sub-endothelial connective tissue in the tunica intima layer have a typical structure. In addition, it is observed that the tunica media layer and tunica adventitia layer have a regular structure. (**C**) (×20) and (**D**) (×40): Sham group: In the aortic root tissue sections of the sham group, subendothelial widespread adipocyte accumulations (curved arrow) are observed in the tunica intima. In addition, adipocyte accumulations (curved arrow) are observed occasionally in the tunica media. (**E**) (×20) and (**F**) (×40): Case group: Dense adipocyte accumulations (curved arrow) are observed in the subendothelial and tunica media in the aortic sections of the case group, (**G**) (×20) and (**H**) (×40): WEP group: In the aortic root sections of the WEP administration group, it is observed that adipocyte accumulations (curved arrow) in the subendothelial and tunica media have decreased. (**I**) (×20) and (**J**) (×40): EEP group: It is observed that adipocytes have decreased in number (curved arrow) in the aortic sections of the EEP administration group. (**K**) (×20) and (**L**) (×40): GW group: In the aortic root sections of the GW administration group, it is observed that adipocyte accumulations in the tunica intima and tunica media layers have decreased (curved arrow). (**M**) (×20) and (**N**) (×40): DMSO group: In the aortic root sections of the DMSO administration group, dense adipocyte accumulations (curved arrow) are observed, especially in the subendothelial layer of the tunica intima and the tunica media layers. (**O**) (×20) and (**P**) (×40): Ethanol group: In the aortic root sections of the ethanol administration group, dense adipocyte accumulations (curved arrow) are observed, especially in the subendothelial layer of the tunica intima and the tunica media layers.

**Figure 6:**
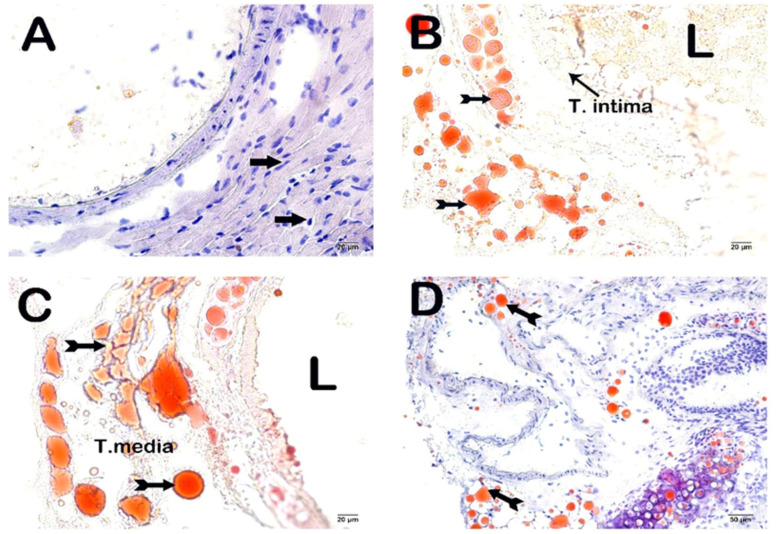

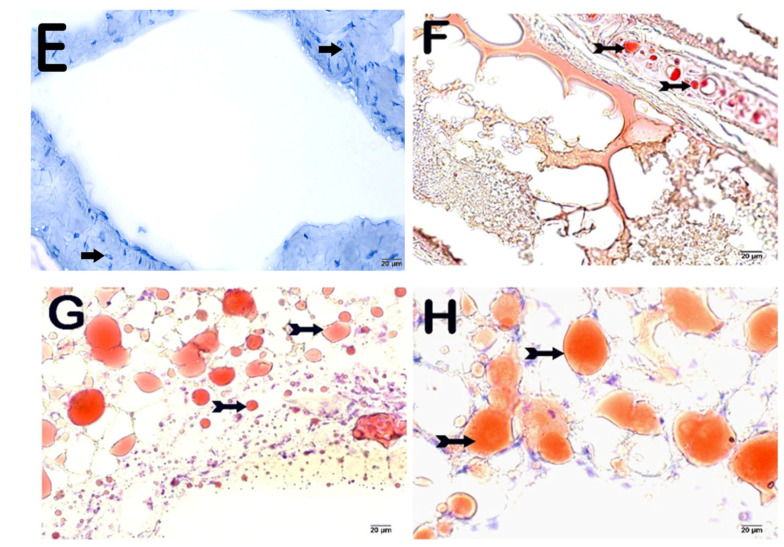
Representative light microscopic image of sections of aortic root tissue stained with ORO. (L: lumen). (**A**) (×10): Control group: In the aortic root sections of the control group, it is observed that the endothelium and subendothelial connective tissue in the tunica intima layer have a typical structure (arrow). In addition, it is observed that the tunica media layer and tunica adventitia layer have a regular structure. (**B**) (×10): Sham group: In the aortic root tissue sections of the sham group, subendothelial widespread adipocyte accumulations (tailed arrow) are observed in the tunica intima. In addition, adipocyte accumulations (tailed arrow) are observed occasionally in the tunica media. (**C**) (×10): Case group: Dense adipocyte accumulations (tailed arrow) are observed in the subendothelial and tunica media in the aortic sections of the case group. (**D**) (×10): WEP group: In the aortic root sections of the WEP administration group, it is observed that adipocyte accumulations (tailed arrow) in the subendothelial and tunica media have decreased. (**E**) (×10): EEP group: It is observed that adipocytes have decreased in number (arrow) in the aortic sections of the EEP administration group. (**F**) (×10): GW group: In the aortic root sections of the GW administration group, it is observed that the adipocyte accumulations in the tunica intima and tunica media layers have decreased (tailed arrow). (**G**) (×10): DMSO group: Dense adipocyte accumulations (tailed arrow) are observed in the aortic sections of the DMSO administration group, especially in the subendothelial layer of the tunica intima and the tunica media layer. (**H**) (×10): Ethanol group: Dense adipocyte accumulations (tailed arrow) are observed in the aortic sections of the ethanol administration group, especially in the subendothelial layer of the tunica intima and the tunica media layer.

In ApoE^-/-^ mice fed with HCD, there was a noticeable increase in atherosclerotic plaque formation and lipid accumulation beneath the endothelial layer compared to the control group (*p* < 0.05). However, after the administration of intraperitoneal GW and propolis extracts for four weeks, the plaque burden and lipid accumulation significantly decreased (*p* < 0.05) (Figure 5 and Figure 6 and Table 3).

### 3.6. Propolis Extracts and GW280264X Lower ADAM10 and Sortilin Levels in the Liver, Improving Dyslipidemia and Non-Alcoholic Fatty Liver Disease

**Figure 7:**
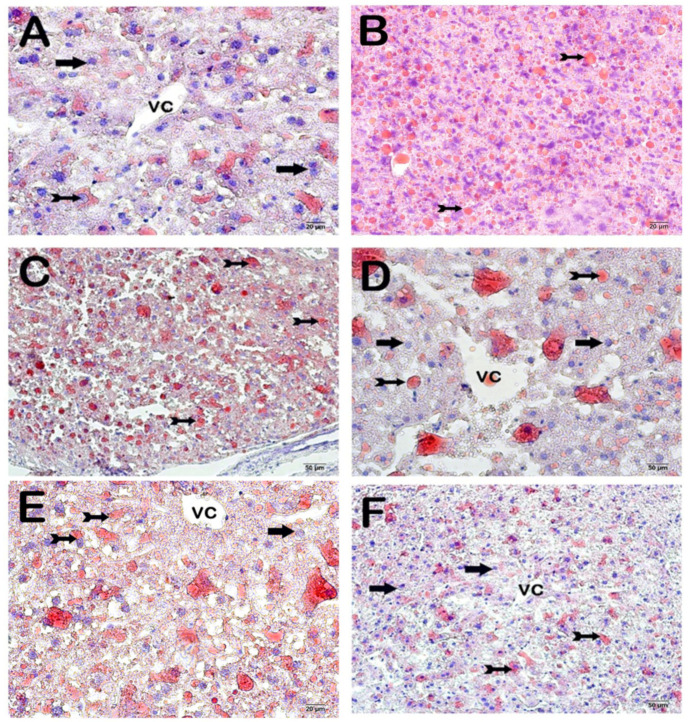

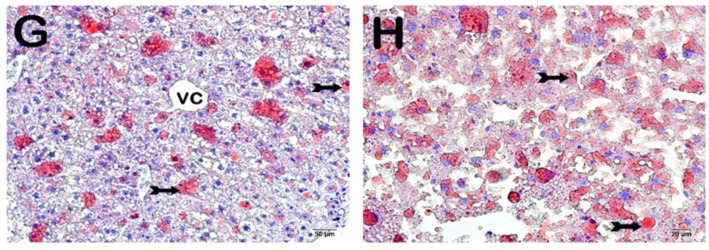
Representative light microscopic image of liver tissue sections stained with ORO. (**A**) (×10): Control group: Observed with remark cords consisting of hepatocytes with typical structure (arrow). Sinusoids are observed between the remark cords (tailed arrow). (**B**) (×10): Sham group: Multifocal hepatic steatosis is observed, formed by degenerative hepatocytes containing numerous lipid droplets and centrally located nuclei. Degenerative hepatocytes containing numerous lipid droplets and centrally located nuclei are observed to have centrilobular and periportal zone involvement (tailed arrow). (**C**) (×10): Case group: Hepatic steatosis (tailed arrow) in the multifocal centrilobular and periportal zones formed by degenerative hepatocytes that commonly contain lipid vacuoles in their cytoplasm. (**D**) (×10): WEP group: It is observed that multifocal steatosis caused by degenerative hepatocytes containing lipid vacuoles in their cytoplasm has decreased (tailed arrow). Hepatocytes (arrow). (**E**) (×10): EEP group: Degenerative hepatocytes containing cytoplasmic lipid vacuoles causing hepatic steatosis are observed to be decreased in the centrilobular and periportal zones (tailed arrow). Hepatocytes (arrow). (**F**) (×10): GW group: Although it is observed that the degenerative hepatocytes that cause multifocal hepatic steatosis have decreased (tailed arrow), hepatocytes with a typical structure are observed (arrow). (**G**) (×10): DMSO group: Hepatic steatosis (tailed arrow) is observed in the multifocal centrilobular and periportal zones formed by degenerative hepatocytes that commonly contain cytoplasmic lipid vacuoles. (**H**) (×10): Ethanol group: Degenerative hepatocytes (tailed arrow) with diffuse cytoplasmic vacuole content causing hepatic steatosis in the centrilobular and periportal zones with a multifocal pattern are observed in the remark cords.

**Figure 8:**
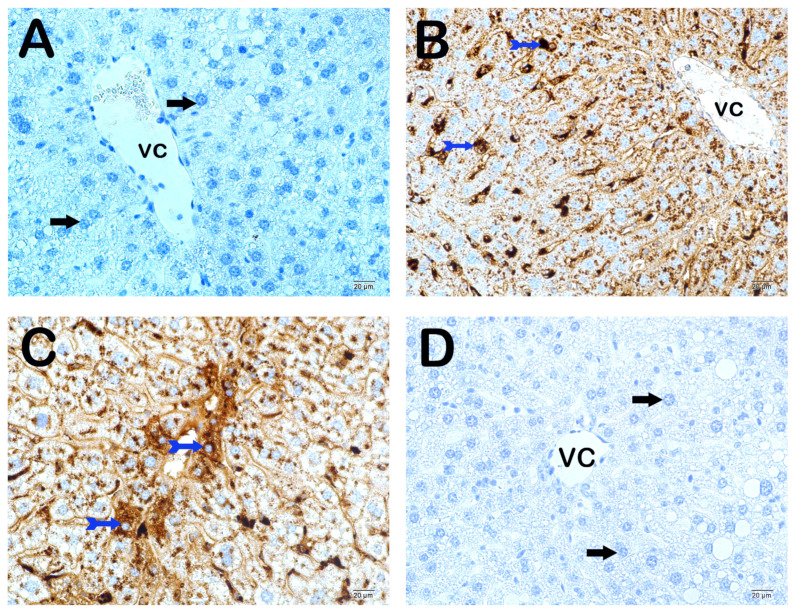

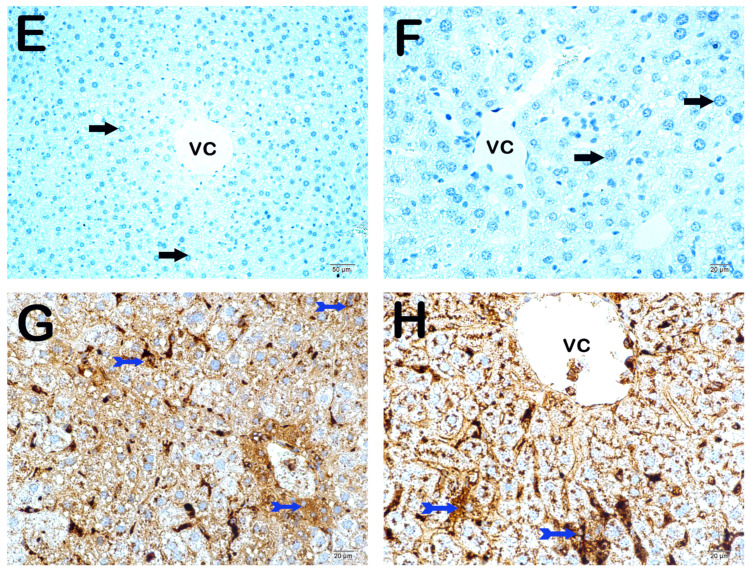
Representative light microscopic picture of liver tissue sections incubated with ADAM10 primary antibody. (**A**) (×10): Control group: Liver tissue consisting of immune-negative normal-structured hepatocytes (arrow) is observed in the liver tissue sections of the control group. (**B**) (×10): Sham group: In the liver tissue sections of the sham group, it is observed that there are a large number of hepatocytes showing intense ADAM10 immune positivity (tailed arrow) in the remark cords. (**C**) (×10): Case group: In the liver tissue sections of the case group, intense ADAM10 immune positivity is observed in many hepatocytes (tailed arrow), especially in the centrilobular region. (**D**) (×10): WEP group: In the liver tissue sections of the WEP administration group, it is observed that the number of hepatocytes showing ADAM10 immune positivity has decreased (arrow). (**E**) (×10): EEP group: In the liver tissue sections of the EEP administration group, it is observed that the number of ADAM10 immune-positive hepatocytes in the remark cords has decreased (arrow). (**F**) (×10): GW group: In the liver tissue sections of the GW administration group, it is observed that the number of ADAM10 immune-positive hepatocytes in the remark cords has decreased (arrow). (**G**) (×10): DMSO group: It is observed that liver tissue sections belonging to the DMSO group contain hepatocytes showing intense ADAM10 immune positivity (tailed arrow) in the perizonal areas, especially in the centripetal region. (**H**) (×10): Ethanol group: It is observed that there are many hepatocytes showing intense ADAM10 immune positivity (tailed arrow), especially in the remark cords of the liver tissue sections of the ethanol group.

**Figure 9:**
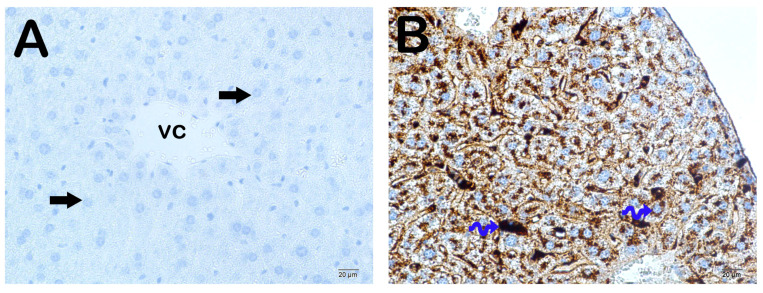

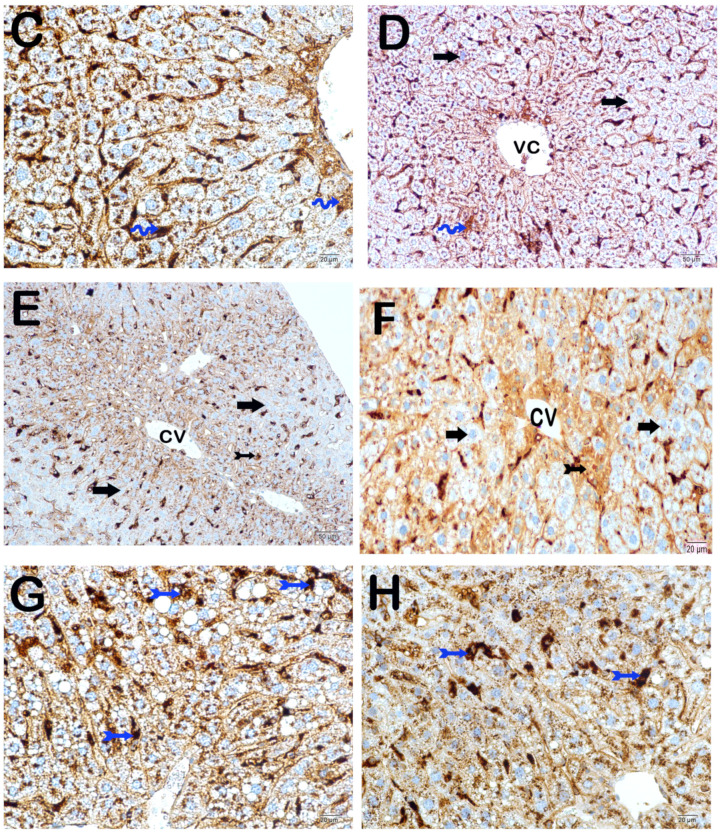
Representative light microscopic picture of liver tissue sections incubated with sortilin primary antibody. (**A**) (×10): Control group: Liver tissue consisting of immune-negative hepatocytes (arrow) is observed in the liver tissue sections of the control group. (**B**) (×10): Sham group: It is observed that there are a large number of hepatocytes (spiral arrow) showing intense sortilin immune positivity in the liver tissue sections of the sham group. (**C**) (×10): Case group: In the liver tissue sections of the case group, intense sortilin immune positivity (spiral arrow) is observed in many hepatocytes in the remark cords. (**D**) (×10): WEP group: In the liver tissue sections of the WEP administration group, it is observed that the number of sortilin immune-positive hepatocytes (spiral arrow) in the remark cords has decreased (arrow). (**E**) (×10): EEP group: In the liver tissue sections of the EEP administration group, it is observed that hepatocytes showing sortilin immune positivity (tailed arrow) have decreased (arrow). (**F**) (×10): GW group: In the liver tissue sections of the GW administration group, it is observed that the number of hepatocytes showing sortilin immune positivity (tailed arrow) in the remark cords has decreased (arrow). (**G**) (×10): DMSO group: It is observed that liver tissue sections belonging to the DMSO group contain hepatocytes showing intense sortilin immune positivity in the perizonal areas, especially in the centripetal region (arrow). (**H**) (×10): Ethanol group: It is observed that there are many hepatocytes showing intense sortilin immune positivity, especially in the remark cords of the liver tissue sections belonging to the ethanol group (arrow).

HCD-induced fatty liver, sortilin, and ADAM10 levels were significantly decreased in the WEP, EEP, and GW groups compared to the case group (*p* < 0.05) (Figure 7, Figure 8 and Figure 9 and Table 4 and Table 5).

## 4. Discussion

Atherosclerosis is a complex health condition that involves chronic inflammation, oxidative stress, and dyslipidemia. Over time, LDL particles and fibrous elements accumulate in large and medium-sized arteries, leading to atherosclerotic plaque formation [2]. Research has shown that inflammation and dyslipidemia play critical roles in developing this condition [25]. To combat these factors, scientists have explored a variety of synthetic and non-synthetic agents, including anakinra [26], methotrexate [27], colchicine [28], statin [29], and propolis [30]. However, previous studies did not focus on ADAM proteases, which are essential regulators of molecules involved in the pathology of atherosclerosis, particularly ADAM10 and ADAM17. In this study, we investigated the effect of the synthetic ADAM10/17 selective inhibitor GW, known for its anti-inflammatory properties, and propolis on atherosclerosis. Our findings suggest that both of these agents have anti-atherogenic activity and that propolis may reduce ADAM10/17 activities, contributing to its efficacy in treating atherosclerosis.

This study was performed in HCD-fed ApoE^-/-^ mice by establishing an atherosclerosis model. Our study found that atherogenic parameters significantly increased in the case group. These included increased BW, TC, TG, Glu, atherosclerotic plaque burden, atherogenic cytokine levels, ADAM10, sortilin, and subendothelial lipid accumulation. Additionally, the HCD diet increased the activity of ADAM10 and 17 in the case group (*p* < 0.05). In line with the existing literature, our data demonstrate that the HCD diet effectively induced atherosclerosis in our experimental groups [31,32]. The increased weight in HCD-induced ApoE^-/-^ mice after WEP, EEP, and GW treatment resulted in a weight loss of 7.6%, 4.2%, and 5.3%, respectively, at the end of the 16th week, showing a protective effect against obesity and obesity-related complications (*p* < 0.01). Ichi et al. reported that ethanol propolis administration prevented weight gain due to decreased adipose tissue mass in rats with high-fat-diet-induced obesity [33]. Koya-Miyata et al. reported that intragastric ethanol propolis supplementation in C57BL/6J mice fed a high-fat diet downregulated the expression levels of sterol regulatory element-binding protein 1/2 and, accordingly, inhibited fatty acid and cholesterol synthesis and reduced visceral fat accumulation [34]. No studies on the effect of WEP and GW on animal weight were found in the literature. Weight loss could occur due to decreased TNF-α levels and ADAM10/17 inhibition [35]. Guan et al. reported that propolis increased the translocation of glucose transporter 4 (GLUT4) to the membrane in skeletal muscle in an STZ-induced diabetes model in C57BL/6J mice fed a high-fat diet, and propolis treatment decreased blood glucose levels [36]. There is no study in the literature investigating the effect of GW on blood glucose levels. However, it may have affected blood glucose levels via two pathways; the first was the downregulation of molecules such as TNF-α, IL-6, and preadipocyte factor 1, just like propolis, and the other was the induction of GLUT4 translocation to the membrane by DMSO, the solvent of GW [37,38]. Rats fed pellets containing Brazilian propolis (0.05−0.5%) showed a dose-dependent decrease in plasma TG and TC [33]. On the other hand, oral administration of Croatian propolis ethanol extract to C57BL/6N mice (50 mg/kg/day) for 30 days reduced serum TG (~11%), TC (~19%), and LDL-C (~35%) [39]. Intraperitoneal injections of ethanolic propolis extract (100 mg/kg, twice a week for 12 weeks) slightly reduced TC levels in ob/ob mice but did not affect TG levels [40].

A study on microparticles isolated from human atherosclerotic plaques reported that ADAM17 increased TNFα, TNF receptor (TNFR), and endothelial protein C receptor levels and induced inflammation and atherosclerotic plaque formation [41]. In addition, it has been reported that the release of inflammatory cytokines in endothelial cells may be caused by the NOTCH signaling pathway activated by ADAM10 and ADAM17 activity [42]. When the serum IL-1β, PLA2, and PON-1 results were examined, it was found that atherogenicity decreased due to the decrease in ADAM10/17 activity. A comprehensive clinical study called CANTOS demonstrated the efficacy of a monoclonal IL-1β inhibitor in treating atherosclerosis [43]. ADAM10/17 is responsible for the sheddase of two key molecules in the IL-1β signaling pathway. With the administration of GW, IL-1R activity was inhibited, and the feedback mechanism was impaired. Propolis extracts were predicted to interrupt the IL-1β signaling pathway by inhibiting ADAM10/17 activity. In addition, they may have reduced the proinflammatory cytokine production signal transmitted by LDL-C, which accumulates and oxidizes in the intima region with its antioxidant activity [44,45,46,47]. Rupture-prone plaques have high levels of PLA2, and PLA2 is known to be released from these plaques into the circulation [48]. No study in the literature shows a direct relationship of propolis or ADAM10/17 with PLA2. In the GW group, the migration of macrophages increased with the inhibition of ADAM10/17 and PLA-2 activity, or the differentiation of macrophages may have favored M2 macrophages. In addition, the synthetic inhibitor used may show inhibitory activity against various MMPs, thus preventing the vicious circle that starts with the effect of PLA-2 and ends with the production of PLA-2. When the aortic arch TNF- α, IFN-γ, IL-6, MPO, IL-12, and IL-10 results were examined, it was found that atherogenicity decreased due to the decrease in ADAM10/17 activity. TNF-α, IL-6, and IL-12 increased ADAM17 activity in the aortic arch during atherosclerosis; ADAM17 activity was statistically significantly lower in the WEP, EEP, and GW groups than in the case group (*p* < 0.05). There was no statistically significant difference between the WEP and GW groups and the control group (*p* > 0.05). In the literature, many studies show that propolis downregulates TNF-α levels [49,50]. Several studies have shown that propolis and its flavonoids can reduce the expression and levels of IL-6 in the body [51,52]. ADAM10/17 is responsible for the sheddase of IL-6R and gp130, an essential part of the IL-6 signaling pathway. Therefore, it is estimated that the decreased IL-6 levels in the GW, WEP, and EEP groups were caused by the decreased activity of these enzymes [53]. MPO, especially HOCl, is known to be involved in the pathophysiology of atherosclerotic diseases [54,55]. The MPO we measured in tissue was statistically significantly lower in the WEP group compared with the case group (*p* < 0.003). Li et al. reported that quercetin, one of the flavonoid components of propolis, inhibited MPO in ApoE^-/-^ mice [56].

It was observed that ADAM10 and ADAM17 activity decreased in both WEP and EEP in the aortic arch compared to the case group; therefore, the results were unsurprising. Although there is ample evidence supporting the role of proinflammatory cytokines in atherosclerosis, the potential role of anti-inflammatory cytokines in this situation is still unknown [57]. IL-10, secreted by Th2 subtype lymphocytes and M2 macrophages, is an anti-inflammatory cytokine [58,59]. IL-10 is involved in many cellular processes that may play an important role in plaque development, for example, inhibition of nuclear factor-kB activation [60,61], metalloproteinase production [62], and cyclooxygenase-2 expression [63], in addition to preventing thrombus formation [64,65]. IL-10, which we measured in tissue, was statistically significantly higher in WEP, EEP, and GW groups compared with the case group (*p* < 0.003).

Histopathologic examinations of the aortic root revealed atherosclerotic plaque burden and subendothelial lipid accumulation. In addition, ADAM10, sortilin, and steatosis levels were investigated histopathologically and immunohistochemically in the liver. It was determined that atherosclerotic plaque burden (H&E staining) and subendothelial lipid accumulation (ORO staining) in the aortic root showed a statistically significant decrease in the WEP, EEP, and GW groups compared to the case group (*p* < 0.05). Fang et al. reported that ethanolic propolis treatment administered by oral gavage at 160 mg/kg/day reduced atherosclerotic plaque burden [30]. The results of lipid accumulation in the aortic root were also supported by the results of the liver, which is the main metabolic organ. The liver tissue section histology was semi-quantitatively (ORO) examined. A statistically significant decrease was observed in the number of degenerative hepatocytes, centrotubular, perizonal, and edematous areas in the WEP, EEP, and GW groups compared to the case group (*p* < 0.05). Nakamura et al. reported that an ethanolic propolis extract was protective against liver damage in rats [66]. Sortilin is a transmembranous protein synthesized in various tissues, especially in the liver, and ADAM10 controls its activity. Sortilin affects hepatic lipoprotein metabolism due to its strong interaction with ApoB100, one of the main apolipoproteins of VLDL [67,68,69]. While numerous studies claim that sortilin increases VLDL secretion, some researchers suggest the opposite. Li et al. reported reduced hepatic lipid accumulation in SORT1^-/-^ mice fed a high-cholesterol diet [70]. Kjolby et al. suggests that SORT1^-/-^ mice secrete less ApoB100 from hepatocytes because a lack of sortilin impairs formation, inhibiting hepatocellular VLDL transport and secretion [67,71]. Gustafsen et al. suggested that overexpression of sortilin causes an increase in proprotein convertase subtilisin/kexin type 9 levels and decreases LDLR levels in hepatocytes [72]. Because of these roles, much evidence suggesting that sortilin promotes atherosclerotic development has been presented [73,74]. However, some results suggest that overexpression of sortilin lowers plasma lipid concentration [75]. As a result of the immunohistochemical examination, ADAM10, and sortilin levels in the liver, there was a statistically significant decrease in WEP, EEP, and GW groups compared to the case group (*p* < 0.05).

## 5. Conclusions

In this study, propolis extracts were shown to reduce dyslipidemia, inflammation, and atherosclerotic plaque burden by reducing ADAM10/17 activity in atherosclerosis development. Propolis may be considered a natural product with potentially promising therapeutic effects in treating atherosclerosis. However, more comprehensive molecular preclinical studies are recommended before clinical applications.

## Figures and Tables

**Table 1:** In silico results.

Receptor Name	Ligand Name	Binding Energy (kcal/mol)	Ki
ADAM10 Extracellular Domain (Chain: A) EC: 3.4.24.81	Pinocembrin	−8.05	1.26 µM
	Caffeic Acid	−6.04	37.6 µM
	Chlorogenic Acid	−6.96	7.93 µM
	Quercetin	−8.07	1.21 µM
	Caffeic Acid Phenethyl Ester	−8.38	716.06 µM
	Trans-cinnamic Acid	−5.49	94.68 µM
	Myricetin	−7.91	1.59 µM
	Galangin	−8.34	776.6 µM
	Chrysin	−8.03	1.31 µM
	Naringenin	−8.45	635.9 nM
	Kaempferol	−8.59	505.9 nM
	Reference Molecule *	−4.49	306.5 nM
ADAM17 Membrane Proximal Domain EC: 3.4.24.86	Pinocembrin	−5.30	129.85 µM
	Caffeic Acid	−4.64	398.29 µM
	Chlorogenic Acid	−5.21	152.67 µM
	Quercetin	−5.11	178.68 µM
	Caffeic Acid Phenethyl Ester	−5.16	164.77 µM
	Trans-cinnamic Acid	−4.77	316.48 µM
	Myricetin	−4.84	282.89 µM
	Galangin	−5.65	72.29 µM
	Chrysin	−5.38	113.77 µM
	Naringenin	−5.54	86.58 µM
	Kaempferol	−5.44	103.74 µM
	Reference Molecule *	−4.99	21.99 µM

*: GW280264X (C_28_H_41_N_5_O_6_S).

**Table 2:** Serum biochemistry results.

Parameters Med (Q1–Q3)	Control	Sham	WEP	EEP	GW	Case	DMSO	Ethanol
	WT (CD)	WT (HCD)	ApoE^-/-^ (HCD)	ApoE^-/-^ (HCD)	ApoE^-/-^ (HCD)	ApoE^-/-^ (HCD)	ApoE^-/-^ (HCD)	ApoE^-/-^ (HCD)
Glu (mg/dL)	162 (161–168)	116 ^a, b^ (109–119)	154 ^a, b^ (152–160)	118 ^a, b, c^ (116–122.5)	133 ^a, b^ (123–143)	185 ^a^ (180–186)	116 ^a, b, c^ (115–118)	115 ^a, b, c^ (109–122)
TG (mg/dL)	76 (72–82)	72 ^b^ (70–79)	54 ^a, b^ (52–58)	57 ^a, b^ (54.5–62)	57 ^a, b^ (51–62.5)	114 ^a^ (110–117)	127 ^a^ (120.8–132)	97 ^a^ (92.3–101.3)
TC (mg/dL)	136 (128–154)	134 (129–138)	1226 ^a, b^ (1212–1267)	1246 ^a, b^ (1103–1259)	1285 ^a, b^ (1253–1326)	1369 ^a^ (1355–1412)	1350 ^a^ (1318–1404)	1410 ^a^ (1327–1505)
ALT (U/L)	42 (38–44)	42 (36–46)	20 ^a, b^ (19–22)	40 (36–50)	34 (32–38.5)	36 (30–42)	25 ^a, b^ (24–26)	35 (34–37)
AST (U/L)	154 (152–154)	252 ^a, b^ (228–254)	142 ^a, b^ (141–147)	280 ^a, b, c^ (275–280)	213 ^a, b, c^ (202.5–217)	163 (161.5–166)	143 (138–147)	240.5 ^a^ (220–265)
BUN (mg/dL)	28 (24–30)	34 (31–34)	30 (26–32)	28 (28–28.5)	28 (22–34)	28 (27–28.5)	35 (34–36.5)	43.5 (41.5–46.8)
IL-1β (ng/mL)	15.04 (14.45–19.3)	50.65 ^a, b^ (32.47–5523)	32.63 ^b^ (18.84–35.9)	43.08 ^a, b^ (42.5–49.2)	32.41 ^a, b^ (30.5–42.81)	63.05 ^a^ (57.89–77.34)	63.05 ^a^ (61.34–65.77)	65.77 ^a^ (62.93–70.83)
PLA2 (ng/mL)	94.81 (90.4–96.94)	160.8 ^a, b^ (145.7–176)	269.8 ^a, b^ (230.2–273.2)	103 ^b, c^ (92.81–113.1)	131.5 ^a, b, c^ (129–145.3)	433 ^a^ (350.2–474.1)	419.5 ^a^ (373.6–458.2)	399.1 ^a^ (357.6–469.1)
PON1 (pg/mL)	621 (564.3–780.3	655.4 ^b^ (560–720.7)	441 ^a, b^ (351.7–446.1)	271 ^a, b, c^ (262.7–291.4)	199 ^a, c^ (160.7–212.5)	142.8 ^a^ (122.7–151.2)	151.3 ^a^ (143.4–167.2)	107.7 ^a^ (106–123.9)

^a^ statistically significant compared to control (*p* < 0.003); ^b^ statistically significant compared to case (*p* < 0.003); ^c^ statistically significant compared to WEP (*p* < 0.003); (*n* = 8, Mann–Whitney U, the data are expressed as median (Q1–Q3). WT: wild-type mice, ApoE^-/-^: Apolipoprotein-E knockout mice, CD: control diet, HCD: high-cholesterol diet.

**Table 3:** Aortic root H&E and ORO scores (ACA/AHA plaque load scoring).

Groups	Aortic Root H&E Score [Median (Q1–Q3)]	Aortic Root ORO Score [Median (Q1–Q3)]
Control	[0 (0–0)]	[0 (0–0)]
Sham	[2 (2–2)] ^a^	[2 (2–2)] ^a^
WEP	[1 (0.5–1)] ^b^	[1 (0.5–1)] ^b^
EEP	[0.5 (0–1)] ^b^	[0.5 (0–1)] ^b^
GW	[1 (0–1)] ^b^	[1 (0–1)] ^b^
Case	[2 (2–3)] ^a^	[2 (2–3)] ^a^
DMSO	[2 (2–2.5)] ^a^	[2 (2–2.5)] ^a^
Ethanol	[2 (2–2.5)] ^a^	[2 (2–2.5)] ^a^

*n* = 8, ^a^ compared to the control group (*p* < 0.05), ^b^ compared to the case group (*p* < 0.05). ACA: American College of Cardiology; AHA: American Heart Association.

**Table 4:** Liver ORO scores (liver histopathological damage score).

Groups	Liver ORO Score [Median (Q1–Q3)]
Control	[0 (0–0)]
Sham	[9 (8–10.5)] ^a^
WEP	[5(3–6)] ^b^
EEP	[3 (2–3)] ^b^
GW	[4 (3.5–5)] ^b^
Case	[9 (7–10)] ^a^
DMSO	[2 (2–2.5)] ^a^
Ethanol	[2 (2–2.5)] ^a^

*n* = 8, ^a^ compared to the control group (*p* < 0.05), ^b^ compared to the case group (*p* < 0.05).

**Table 5:** Liver ADAM10 and sortilin positivity scores.

Groups	Liver ADAM10 [Median (Q1–Q3)]	Liver Sortilin Score [Median (Q1–Q3)]
Control	[0 (0–0)]	[0 (0–0)]
Sham	[3 (2–3)] ^a^	[2 (2–3)] ^a^
WEP	[1 (1–1.5)] ^b^	[1 (1–1.5)] ^b^
EEP	[1 (1–1)] ^b^	[1 (1–1)] ^b^
GW	[1 (1–1)] ^b^	[1 (1–1)] ^b^
Case	[3 (3–3)] ^a^	[2 (2–3)] ^a^
DMSO	[2 (2–3)] ^a^	[2 (2–3)] ^a^
Ethanol	[3 (2–3)] ^a^	[3 (2–3)] ^a^

*n* = 8, ^a^ compared to the control group (*p* < 0.05), ^b^ compared to the case group (*p* < 0.05).

## Data Availability

All data generated or analyzed during this study are included in this published article.
